# The combination treatment of oncolytic adenovirus H101 with transcatheter arterial embolization sequential thermal ablation for hepatocellular carcinoma: a retrospective study

**DOI:** 10.3389/fonc.2025.1623877

**Published:** 2025-09-19

**Authors:** Changyou Jing, Tong Zhu, Yonghong Zhang, Jianjun Li

**Affiliations:** Hepatic Disease and Oncology Minimally Invasive Interventional Center, Beijing Youan Hospital, Capital Medical University, Beijing, China

**Keywords:** hepatocellular carcinoma, oncolytic adenovirus, H101, transcatheter arterial embolization, thermal ablation

## Abstract

**Background & Aims:**

Recombinant human adenovirus type 5 (H101) was demonstrated to enhance the efficacy of transcatheter arterial embolization (TAE) for hepatocellular carcinoma (HCC). This study aims to analyze the efficacy and safety of oncolytic adenovirus H101 combined with TAE sequential thermal ablation in HCC.

**Methods:**

This single-center retrospective study evaluated the progression-free survival (PFS) and overall survival (OS) of HCC patients who received H101 combined with TAE sequential thermal ablation therapy from July 2015 to January 2022. Adverse reactions during treatment were recorded.

**Results:**

A total of 55 HCC patients were included, with a median follow-up of 39 months (range: 10–106 months). During the follow-up period, disease progression was observed in 43 of 55 patients, and 22 of the 55 patients died. The median OS and PFS time were 77 and 12.3 months, respectively. The one-, two-, and three-year OS rates were 94.5%, 86.3%, and 77.3%, respectively. The PFS rates at one, two and three years were 56.4%, 26.3% and 20%, respectively. Multivariate analysis revealed that the diameter was independent predictors of PFS (P = 0.023). No patient experienced a serious adverse event, or a fatal or disabling event, due to the injection of oncolytic virus.

**Conclusion:**

This study suggests that HI01 combined with TAE sequential thermal ablation is both safe and effective for HCC, warranting further investigation through prospective randomized controlled trials.

## Introduction

According to Global Cancer Statistics 2022, primary liver cancer (PLC), which remains the third most lethal cancer among all malignant tumors, accounts for 7.8% of all cancer - related deaths, with hepatocellular carcinoma (HCC) making up 75 - 85% of PLC cases (1). Although traditional treatment methods such as hepatectomy, liver transplantation, transcatheter arterial chemoembolization (TACE), radiofrequency ablation (RFA) and microwave ablation (MWA) have been widely used, due to the high heterogeneity and immune escape mechanisms of HCC, a single treatment modality often fails to achieve a durable treatment effect. Moreover, it is important to recognize that the rate of local tumor progression in HCC remains high when relying on a single treatment modality such as surgery, ablation, or TACE. This underscores the necessity for a multimodal and personalized approach in HCC management to enhance patient outcomes and reduce the risk of tumor recurrence and progression (2, 3). TACE is primarily used for HCC cases that are considered inoperable or for patients who are not suitable candidates for surgical resection. Nevertheless, TACE alone often exhibits limited tumor necrosis and is prone to recurrence in residual lesions (4). The combination of TACE and ablation has demonstrated a synergistic effect, leading to more extensive tumor necrosis and improved survival rates compared to monotherapy (5–8). A retrospective study conducted at our institution demonstrated that the 5-year recurrence rate in patients treated with a combination of TACE and ablation was still as high as 80% (9). It is therefore necessary to optimize the current routine treatment of HCC and explore new treatment strategies.

As a novel tumor immunotherapy approach, oncolytic adenovirus H101 can specifically infect and kill tumor cells and simultaneously activate the host’s anti-tumor immune response. H101 induces the immunogenic death of tumor cells through virus replication and tumor cell lysis, thereby enhancing the body’s anti-tumor immune response (10, 11). Previous studies have shown that H101 has demonstrated good preclinical and clinical effects in the treatment of various solid tumors (12–14). In addition, H101 was approved for market release by the China National Medical Products Administration (NMPA) in 2005, some studies have further confirmed that H101 combined with TACE improves the prognosis of patients with HCC (15–17). However, there are currently no relevant literature reports on the treatment of transarterial embolization (TAE) combined with H101 in sequence with thermal ablation. Therefore, in this study, a retrospective analysis was conducted to evaluate the efficacy and safety of H101 combined with TAE in sequence with thermal ablation in the treatment of HCC. The primary endpoints examined in this analysis were the tumor recurrence, progression, and overall survival (OS) status of the patients.

## Materials and methods

### Patient selection

A retrospective analysis was conducted on HCC patients who underwent H101 combined with TAE sequential ablation treatment at Beijing You’an Hospital, Capital Medical University, between July 2015 and January 2022. The inclusion criteria were as follows: a) HCC diagnosis confirmed by magnetic resonance imaging (MRI), computed tomography (CT), digital subtraction angiography (DSA), or tissue pathology. b) Patients who underwent TAE followed by ablation to treat HCC. It is also required that during the TAE procedure, H101 be injected into the tumor-feeding artery through the catheter. c) Patients categorized as Barcelona Clinic Liver Cancer (BCLC) Staging System stages 0, A, or B based on information available in the hospital’s Picture Archiving and Communication Systems (PACS) and Electronic Medical Record System (EMRS). d) All patients underwent follow-up for a minimum of three months after the ablation procedure. The study was in line with the Declaration of Helsinki. This retrospective study has been approved by the Ethics Committee of Beijing You’an Hospital Affiliated to Capital Medical University (LL-2022-130-k).

### Treatment protocols

**Figure 1:**
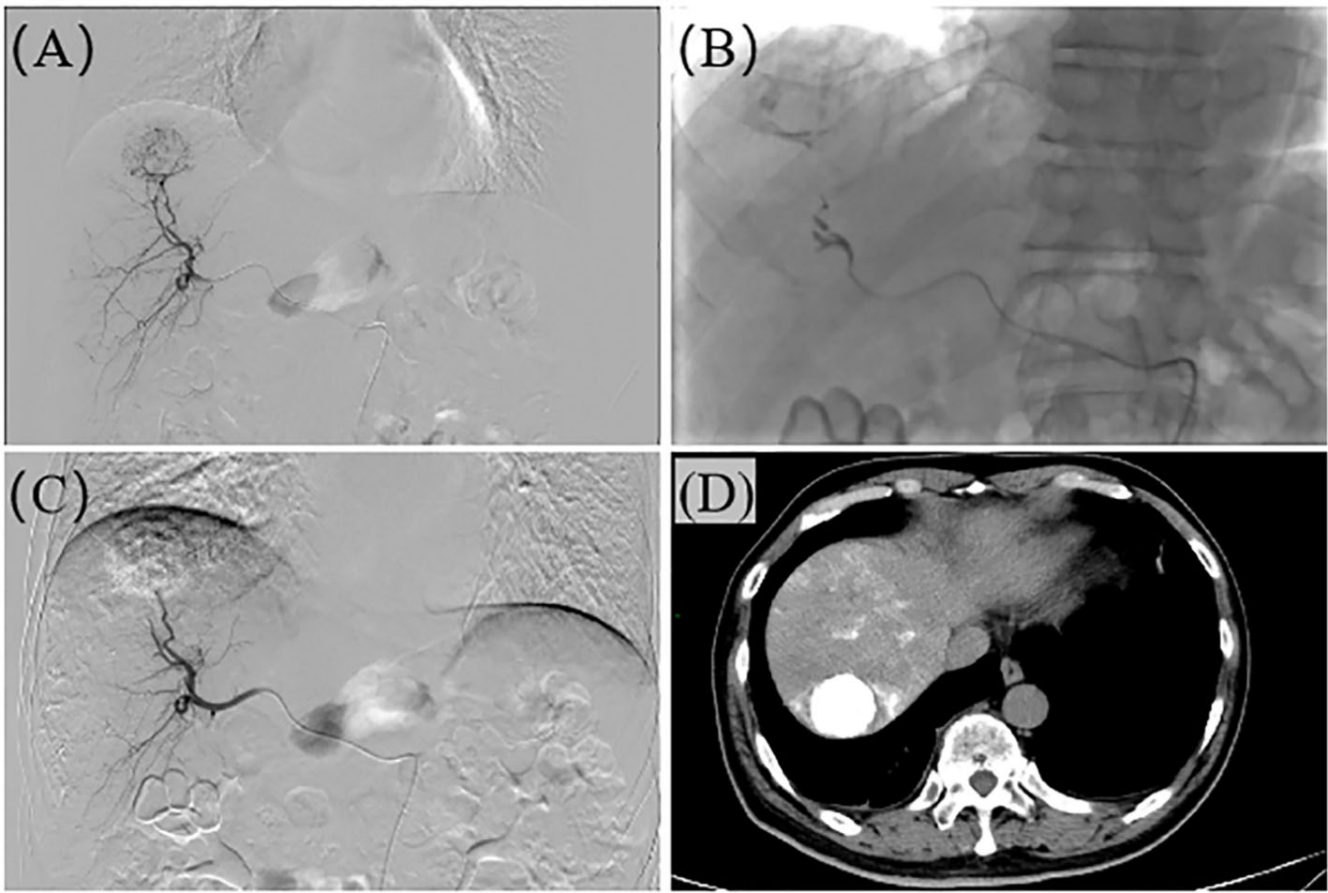
The operation process of TAE. **(A)** Hepatic arteriography reveals a roundish tumor stain in segment S7 of the liver. **(B)** The microcatheter is super-selectively advanced to the tumor-feeding artery, and embolization is performed with 101, lipiodol, and gelfoam respectively. **(C)** After the embolization is completed, re-angiography shows no tumor staining, and the embolic agents are filled within the tumor. **(D)** Plain CT after TAE confirms the presence of roundish lipiodol deposition foci in the liver, with dense lipiodol deposition within the tumor.

**Figure 2:**
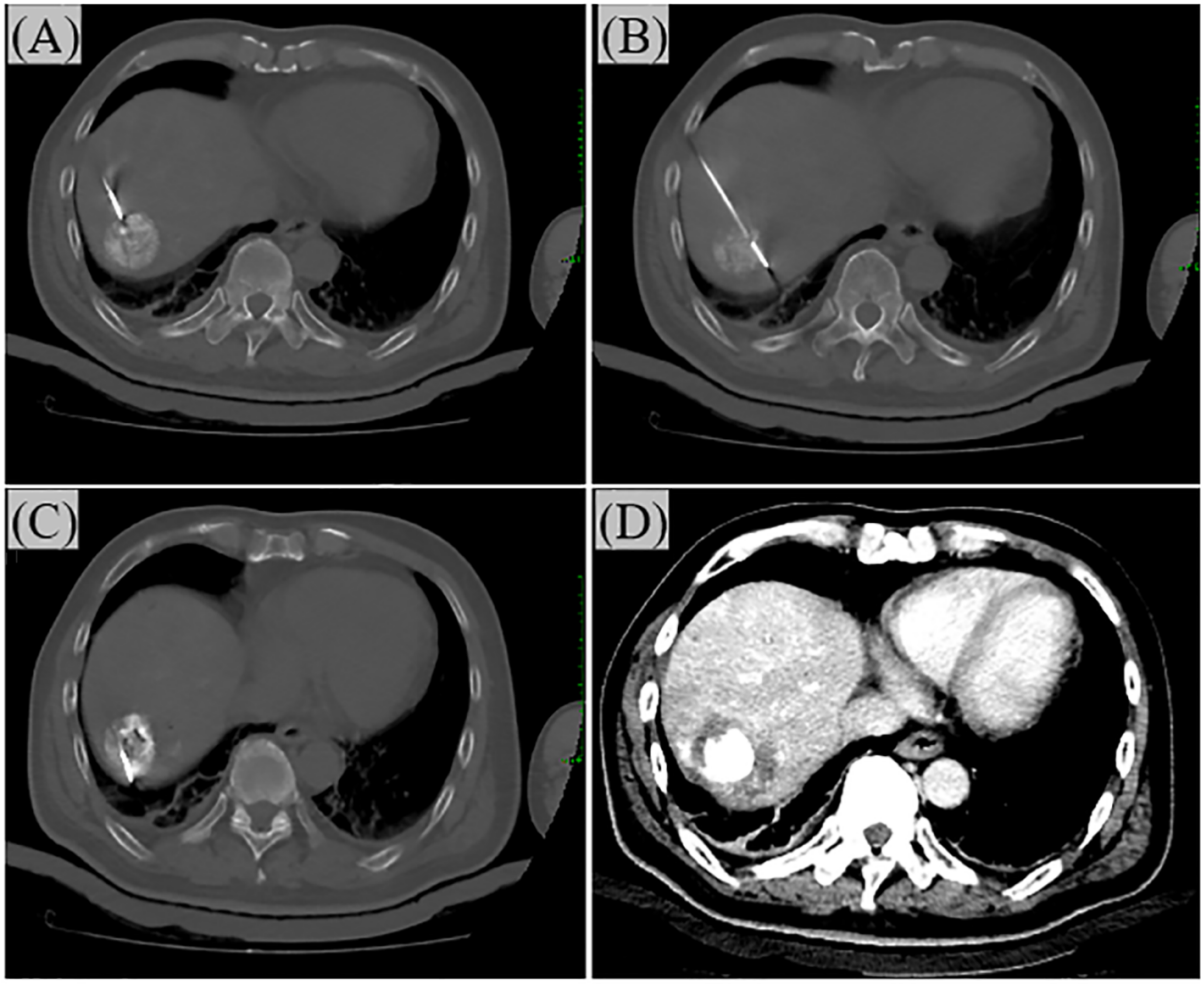
The operation process of Ablation. **(A)** Under CT guidance, the needle is inserted from the ventral side of the tumor. In the figure, the ablation needle has just entered the edge of the tumor. **(B)** Progressing step by step, the ablation needle enters the center of the tumor. **(C)** Insert the needle again to ablate the dorsal side of the tumor. **(D)** After the ablation is completed, perform a CT reexamination to ensure complete ablation. The ablation margin should be at least 0.5 cm.

In the study, all patients initially received TAE as the primary treatment. H101 was administered to all enrolled patients. The specific procedure was as follows: during the TAE operation, after super-selectively inserting a microcatheter into the tumor-feeding artery, H101 was injected first (at a dose of 1.0×10¹² viral particles dissolved in 10 mL of 0.9% normal saline), followed by embolization with lipiodol (Ethiodol; Laboratoires Guerbet, Roissy, France) and the gelatin sponge particles embolization (Ailikang pharmaceutical technology co., LTD., Hangzhou, China). The sterile and purified viral batches, manufactured for clinical application in humans, were supplied by Shanghai Sunway Biotech (Shanghai, China) and underwent testing for potency, sterility, and overall safety at the National Institute for the Control of Pharmaceutical and Biological Products (Beijing, China) (18, 19). The operation process of TAE is shown in **Figure 1**. After TAE, patients typically underwent ablation treatment within a month, within 1–2 weeks after TAE. The main percutaneous ablation techniques applied were RFA or MWA, both performed by skilled attending physicians. Each method operates through a unique mechanism. All ablation procedures were conducted under CT guidance. Generally, RFA was utilized for single tumors smaller than 3 cm, while MWA was preferred for tumors exceeding 5 cm in diameter. Nonetheless, the final selection of the ablation technique was based on the tumor’s size, location, and proximity to major blood vessels, as assessed by the treating physician. The operation process of ablation is shown in **Figure 2**.

### Follow-up

Patient follow-up data were retrieved from PACS and EMRS. All assessments were reviewed independently by two radiologists to reduce subjective bias. Progression-free survival (PFS) was defined as the duration from the initiation of H101 therapy to the occurrence of tumor recurrence, the appearance of new lesions, metastasis, death, or the end of follow-up. Progression was assessed using RECIST 1.1 criteria: Imaging (CT/MRI/DSA) evidence of ≥20% increase in the longest diameter of existing lesions, appearance of new lesions, or distant metastasis. PFS was evaluated using imaging modalities such as CT, MRI, and DSA. Clinical AEs were graded according to the National Cancer Institute (NCI) Common Terminology Criteria for Adverse Events (CTCAE) v5.0 criteria. The follow-up cutoff date was October 31, 2024.

### Statistical analysis

Continuous variables were expressed as mean ± standard deviation (SD), while categorical variables were presented as percentages. The Student’s t-test was employed for comparisons of continuous data, whereas the chi-square test was utilized for categorical data. PFS and OS were estimated using the Kaplan-Meier method, and differences between groups were assessed with Log-rank tests. Univariate and multivariate analyses were performed using the Cox regression model. Variables with P-values less than 0.1 in the univariate analysis were subsequently included in the multivariate analysis. A statistically significant difference was indicated by P-values less than 0.05. Data analysis was carried out using IBM SPSS software version 25.0 (IBM Corp., Armonk, NY).

## Results

### Patients characteristics

We conducted a retrospective analysis of data from 55 patients, comprising 43 men and 12 women with a median follow-up of 39 months (range: 10–106 months). The average age at HCC diagnosis was 63.9 ± 10.0 years, with a range of 41 to 87 years old in our hospital. The primary cause of tumors was cirrhosis (81.8%), followed by hepatitis B virus (HBV) infection alone (70.9%). According to the BCLC staging, there were 32 patients with BCLC stage 0/A(early-stage HCC) and 23 patients with intermediate-stage HCC (BCLC stage B). Additional details of the patients’ characteristics are presented in **Table 1**.

**Table 1:** Clinical characteristics and follow-up results from 55 patients of HCC.

Variables	Total (n=55)
Gender, n (%)	
Male	43 (78.2)
Female	12 (21.8)
Age (years), mean ± SD	63.9 ± 10.0
Family medical history, n (%)	2 (3.9)
Virus infection, n (%)	
None	14 (25.5)
HBV	39 (70.9)
HCV	1 (1.8)
HBC+HCV	1(1.8)
Cirrhosis, n (%)	45 (81.8)
Child-pugh score, n (%)	
A	47 (85.5)
B	8 (14.6)
BCLC Stage, n (%)	
0	6 (10.9)
A	26 (47.3)
B	23 (41.8)
AFP (ng/mL), median (IQR)	5.2 (2.6, 45.8)
≤7	30 (54.6)
>7	25 (45.5)
ALB (g/dL), median (IQR)	37.6 (34.1, 40.2)
TBIL (µmol/L), median (IQR)	21.4 (15.0, 27.1)
Platelet count (10^9^/L), median (IQR)	100.5 (70.0, 147.0)
No. of tumors, n (%)	
1	25 (45.5)
≥2	30 (54.6)
Maximum tumor diameter (cm), n (%)	
≤3	33 (60.0)
>3	22 (40.0)
Progression, n (%)	
Yes	43 (78.2)
No	12 (21.8)
Death, n (%)	
Yes	22 (40.0)
No	33 (60.0)

HCC, hepatocellular carcinoma; HBV, hepatitis B virus; HCV, hepatitis C virus; BCLC, Barcelona Clinic Liver Cancer; AFP, alpha fetoprotein; ALB, serum albumin; TBIL, total bilirubin.

### Outcome and follow-up

**Figure 3:**
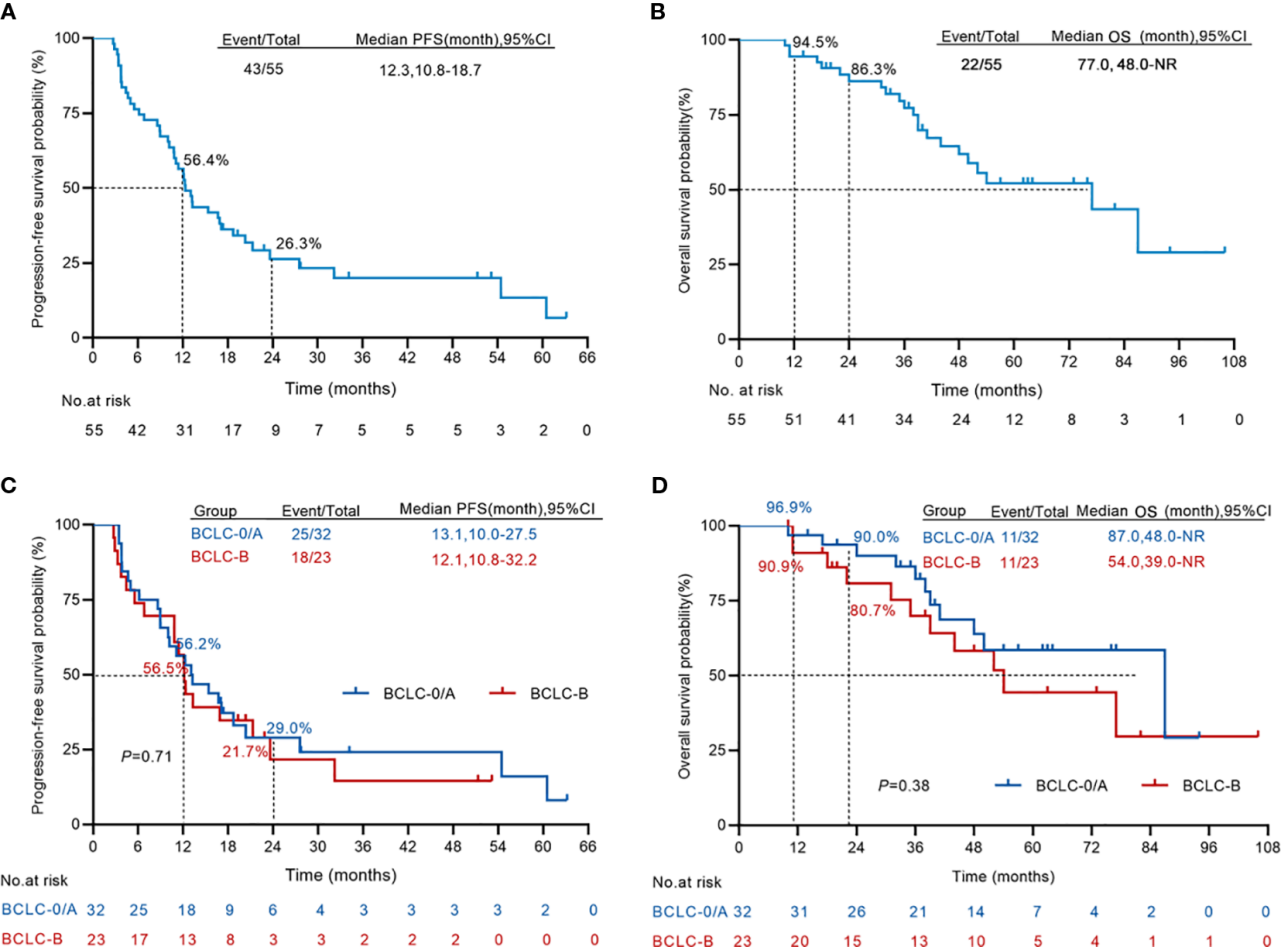
Kaplan–Meier curves for progression-free survival and overall survival. **(A, B)** The Kaplan–Meier survival analysis of overall survival **(A)** and progression-free survival **(B)** from the first H101 therapy (n = 55). **(C, D)** The Kaplan–Meier survival analysis comparing the overall survival **(C)** and progression-free survival **(D)** from the first H101 therapy of early-stage HCC (n = 32) and intermediate-stage HCC (n = 23). NR, not reach.

In the patients of this cohort, disease progression was observed in 43 of 55 patients, and 22 of the 55 patients died. The median OS and PFS time were 77 and 12.3 months, respectively (**Figure 3A, B**). The one-, two-, and three-year OS rates were 94.5%, 86.3%, and 77.3%, respectively. The PFS rates at one, two and three years were 56.4%, 26.3% and 20% respectively. The median PFS of BCLC stage 0/A HCC patients is 13.1 months, while that of intermediate-stage HCC patients is 12.1 months. When it comes to the one-year PFS, the rates are 56.2% and 56.5% for early- and intermediate- stage HCC patients respectively. Looking at the three-year PFS, the percentages are 21.4% and 14.5% for these two groups (**Figure 3C**). The median OS of BCLC stage 0/A patients is 87 months, and for intermediate-stage HCC patients, it is 54 months. The one-year OS rates are 96.9% and 90.9% for early- and intermediate-stage HCC patients respectively. The three-year OS rates are 82.3% and 70.0% for these two stages (**Figure 3D**).

### Univariate and multivariate analyses

Multivariate Cox regression analysis was conducted, including gender, age, family medical history, viral infection, liver cirrhosis, Child Pugh score, BCLC stage, alpha fetoprotein (AFP), tumor number, and maximum tumor diameter (**Table 2**). The univariate analysis demonstrated that the liver cirrhosis, tumor number, and maximum tumor diameter were risk factors for PFS (*P* < 0.05). The risk of progressive disease events among patients with liver cirrhosis was 3.020 times greater than that observed in patients without liver cirrhosis (P = 0.022). The risk of progressive disease events in patients with multiple tumors was 2.407 times higher than in patients with single tumor (P = 0.007). The maximum tumor diameter was positively correlated with progressive disease, and the risk of progressive disease in patients with a large maximum diameter was 2.771 times higher than that in patients with a smaller maximum diameter (P = 0.002). Subsequently, we incorporated liver cirrhosis, tumor number, and maximum tumor diameter into the multivariate analysis. The results showed that the maximum tumor diameter was an independent prognostic factor for progressive disease events (HR = 2.310, P = 0.023). Furthermore, we conducted a Cox regression analysis on OS (**Table 3**). Univariate and multivariate analyses showed that none of these indicators, including group variables, had a statistically significant effect on OS.

**Table 2:** Univariate and multivariate analyses of factors associated with PFS.

Variables	Cases/N	Median PFS (95%CI)	Univariate analysis	Multivariate analysis	
			*P* value	HR (95%CI)	*P* value
Gender					
Male	34/43	12.3 (10.8, 18.7)			
Female	9/12	13.3 (8.6, NR)	0.833		
Age (years)					
<65	19/24	14.5 (10.8, 32.2)			
≥65	24/31	12.3 (10.0, 20.3)	0.638		
Family medical history					
None	12/16	13.3 (8.9,NR)			
Yes	31/39	12.1 (10.2,21.3)	0.771		
Virus infection	31/39	12.1 (10.2, 21.3)	0.882		
HBV	31/38	12.1 (10.0, 20.3)	0.950		
HCV	1/2	60.5 (NR, NR)	0.133		
Child-pugh Score					
A	36/47	13.1 (10.8, 21.3)			
B	7/8	12.2 (8.9, NR)	0.266		
BCLC Stage					
0/A	25/32	13.1 (10.0, 27.5)			
B	18/23	12.1 (10.8, 32.2)	0.711		
Cirrhosis					
None	5/10	32.2 (16.9, NR)		1	
Yes	38/45	11.4 (8.9, 16.7)	**0.022**	2.344 (0.783, 7.108)	0.128
No. of tumors					
1	16/25	21.3 (12.3, NR)		1	
≥2	27/30	10.8 (6.8, 16.7)	**0.007**	1.222 (0.535, 2.790)	0.635
Maximum tumor diameter (cm)					
≤3	23/33	16.7 (12.3, NR)		1	
>3	20/22	8.8 (5.5, 16.9)	**0.002**	2.310 (1.121, 4.762)	**0.023**
AFP (ng/mL)					
≤7	23/30	12.7 (10.8, 18.7)			
>7	20/25	12.1 (8.9, 54.4)	0.642		

NR, Not Reached; HR, Hazard Ratio; CI, confidence interval; HBV, hepatitis B virus; HCV, hepatitis C virus; BCLC, Barcelona Clinic Liver Cancer; AFP, alpha fetoprotein. Bold values indicate P < 0.05.

**Table 3:** Univariate and multivariate analyses of factors associated with OS.

Variables	Cases/N	Median OS (95%CI)	Univariate analysis	Multivariate analysis	
			*P* value	HR (95%CI)	*P* value
Gender					
Male	16/43	77.0 (50.0, NR)			
Female	6/12	41.0 (31.0, NR)	0.436		
Age (years)					
<65	9/24	77.0 (54.0, NR)			
≥65	13/31	52.0 (41.0, NR)	0.554		
Family medical history					
None	20/50	77.0 (48.0, NR)			
Yes	1/2	44.0 (44.0, NR)	0.771		
Virus infection	14/39	77.0 (48.0, NR)	0.176		
HBV	14/38	77.0 (48.0, NR)	0.219		
HCV	0/2	–	0.998		
Cirrhosis					
None	1/10	NR (NR, NR)			
Yes	21/45	52.0 (41.0, NR)	0.110		
Child-pugh Score					
A	18/47	77.0 (50.0, NR)			
B	4/8	39.0 (24.0, NR)	0.179		
BCLC Stage					
0/A	11/32	87.0 (48.0, NR)			
B	11/23	54.0 (39.0, NR)	0.383		
No. of tumors					
1	6/25	NR (52.0, NR)		1	
≥2	16/30	44.0 (38.0, NR)	0.090	1.884 (0.671, 5.289)	0.229
Maximum tumor diameter (cm)					
≤3	11/33	87.0 (50.0, NR)		1	
>3	11/22	44.0 (38.0, NR)	0.097	1.621(0.656,4.009)	0.296
AFP (ng/mL)					
≤7	12/30	54.0 (48.0, NR)			
>7	10/25	77.0 (38.0, NR)	0.910		

### Safety

According to the CTCAE 5.0 criteria, 65% (36/55) of the fever events were grade 1 (body temperature 38.1-39.0°C), with no grade 2 or higher fever (>39°C) observed. All fever events resolved within 24–48 hours after symptomatic treatment. Among ablation-related complications, 100% (55/55) were grade 2 transaminase elevations (Alanine Aminotransferase/Aspartate Aminotransferase 2–5 times the upper limit of normal), which recovered within one week after hepatoprotective treatment, with no grade 3 or higher severe events. No typical immune-related adverse events (irAEs) such as rash, colitis, or thyroid dysfunction were observed caused by H101. (**Table 4**).

**Table 4:** Treatment-related Adverse Event.

Adverse Event	Grade	Incidence, n (%)	Description	Management	Outcome
Fever-related complications					
Fever	1	36 (65.5)	Body temperature 38.1-39.0°C; onset within 24 hours post-TAE.	Antipyretics or physical cooling	Resolved within 24-48 hours
Higher-grade fever (>39°C)	≥2	0	No such events observed.	N/A	N/A
Ablation-related complications					
Transaminase elevation	2	55 (100)	ALT/AST levels 2-5 times the upper limit of normal; detected 3-7 days post-ablation.	Hepatoprotective agents	Recovered within 1 week
Severe liver dysfunction	≥3	0	No grade 3+ events (e.g., ALT/AST >5×ULN or liver failure) recorded.	N/A	N/A
Immune-related adverse events (irAEs)					
Rash, colitis, thyroid dysfunction	N/A	0	No typical irAEs associated with H101 observed during follow-up.	N/A	N/A

TAE, transcatheter arterial embolization; ALT, Alanine Aminotransferase; AST, Aspartate Aminotransferase; ULN, Upper Limit of Normal; irAEs, Immune-related adverse events.

## Discussion

TACE combined with ablation therapy has been reported in many studies to reduce the recurrence rate of patients and improve their prognosis (5, 7, 20). However, according to the early retrospective study reports from our center, the 5-year recurrence rate of TACE combined with ablation still remains as high as 80% (9). Although oncolytic viruses have been reported in many tumors, most of the reports are about the direct injection of H101 into the tumor body (13, 14, 21). In addition, there are very few reports on the efficacy of TACE combined with oncolytic viruses for HCC at present. Whether the chemotherapeutic drugs during hepatic artery embolization affect H101 remains to be further verified. In this study, TAE combined with H101 can avoid the influence of chemotherapeutic drugs on the efficacy of H101. In addition, research has confirmed that oncolytic viruses can enhance anti-tumor immunity and induce pyroptosis of vascular endothelial cells to inhibit tumor growth (12). Ablation therapy can also promote the release of tumor antigens and stimulate the immune response in the body (22). The sequential thermal ablation treatment of TAE combined with H101 is reported for the first time. This combined therapy offers a novel therapeutic strategy for the treatment of HCC.

Although more than 41.8% of patients in this study were non-treatment-naïve, the median 3-year OS for early and intermediate-stage HCC remained higher than those reported in the literature. According to the literature, the median survival time from recurrence to death for patients with HCC undergoing surgical resection is reported to be 21 months (23). Additionally, a study with a slightly larger sample size, involving 497 patients with recurrent HCC, developed a prognostic model to predict survival after recurrence (SAR), demonstrating a median SAR of 41.2 months (24). For patients classified as having intermediate-stage HCC according to the BCLC staging system, this study indicates that the 3-year OS rate is 70%, which similar to the results of our previous retrospective study (9). On one hand, it is believed that the combination of TAE and ablation can improve patient prognosis. On the other hand, in the study, only 4 out of 23 patients had AFP levels greater than 400 ng/ml. Elevated serum AFP levels are associated with poor prognosis in HCC patients (25). This also suggests that H101 may have a better effect on HCC patients who are AFP-negative. The favorable outcomes observed in patients with tumors ≤ 3 cm or AFP-negative status in this study are merely retrospective correlations, and a causal relationship has not been established. These outcomes may be related to confounding factors such as lower tumor burden and more indolent biological behavior in such patients, requiring further verification through prospective studies. Regrettably, the tumor progression rate in this study remains high, with 1-year PFS rates for HCC patients at 56.4%. This is consistent with the majority of literature reports and represents a challenge in the treatment of HCC (26). For example, Yun et al. reported that the cumulative 1-year recurrence rate of patients with early-stage HCC after TACE was 43.9%, while Wang et al. reported that the cumulative 1-, 3-, and 5-year recurrence rates of patients with early-stage HCC after TACE combined locoregional ablation were 26.0% (142/547), 57.8% (316/547), and 68.2% (373/547), respectively (27, 28). However, this does not mean that H101 is of no significance in the treatment of HCC. The reasons are analyzed as follows: First, in this study, nearly 41.8% of the patients had previously undergone surgical resection or multiple TACE or ablation treatments. In addition, 4 patients had undergone transjugular intrahepatic portosystemic shunt (TIPS) treatment due to gastrointestinal bleeding. Although most patients had Child-Pugh class A liver function, this was largely achieved through pharmacological intervention. These patients had undergone multiple treatments, suggesting that they might be at high risk for recurrence or progression. Second, the majority of patients exhibit good compliance; among the patients, 69.1% (38/55) have hepatitis B, which implies that these patients require long-term antiviral treatment and regular follow-ups to monitor viral load and liver function. Third, after ablation, patients are required to attend follow-ups at our hospital for three consecutive months, which allows for the early detection of any new lesions. For patients with BCLC stage B, the median PFS time in this study was 12.1 months. Zhang et al. reported that the median PFS time of patients after transarterial chemoembolization - microwave ablation (TACE-MWA) was also 10 months (29). However, all the patients included in their study were treatment-naive. In contrast, in this study, only 61% (14/23) of the patients with BCLC stage B were treatment-naive, while the remaining patients had received multiple previous local treatments, including surgery, ablation, and TACE. This indirectly indicates that for patients with BCLC stage B, the combination treatment of H101 with TAE and ablation is superior to TACE - MWA in therapeutic efficacy, but further research is needed to confirm this. Additionally, in the present study, all six patients (10.9%) with BCLC stage 0 disease presented with individual clinical factors precluding standard curative therapy: three exhibited severe cirrhosis, for whom the combined modality approach was selected to mitigate procedure-related hemorrhagic risk during ablation; and three had tumors critically abutting major vasculature or biliary structures at the hepatic hilum, where achieving an adequate ablative margin with stand-alone ablation was not feasible. In this cohort, we employed a sequential regimen of H101 combined with TAE followed by thermal ablation. This therapeutic strategy was designed to leverage the synergistic oncolytic, embolic, and ablative effects, thereby reducing procedural invasiveness while enhancing local tumor control.

The characteristics of cirrhosis are diffuse liver fibrosis and the replacement of normal liver structures with regenerated liver nodules, which have been proven to be positively correlated with liver cancer (30). The findings of this study consistently indicate that patients with cirrhosis have a 3.020 times higher risk of progression compared to those without cirrhosis. Moreover, tumor-related factors have been reported to be risk factors for recurrence after curative treatment in patients with early- stage HCC, such as tumor size, number of tumors, and pathological type (28). In this study, we found that the number of tumors and the maximum tumor diameter were risk factors for tumor progression. Furthermore, multivariate analysis showed that only the maximum tumor diameter was an independent prognostic factor for disease progression in HCC patients after H101 combined with TAE sequential thermal ablation. Our results suggest that HCC patients with a maximum tumor diameter of ≤ 3 cm are more likely to benefit from H101 combined with TAE sequential thermal ablation therapy.

In terms of safety, this study did not identify any serious adverse events (SAEs) caused by the injection of H101. The most common complication associated with H101 was fever that was consistent with the reports in the literature (14, 31). However, upon reviewing medical records, we found that such fever typically does not persist beyond 24 hours. Notably, this fever differs from that caused by TAE-induced post-embolization syndrome or infection secondary to tumor necrosis. The primary distinction lies in the timing of onset, as the latter usually occurs more than 24 hours after the procedure. However, strictly speaking, due to the lack of a control group in this study, and the fact that fever and abnormal transaminase levels can also occur after TAE, a randomized controlled study would be better able to distinguish between the two.

This study has several limitations. Firstly, it is a single-center, small-sample retrospective study, introduces multiple potential biases including selection bias, information bias, and confounding factors. Secondly, due to the lack of control group, it is difficult to distinguish whether the observed treatment effects result from H101, TAE, thermal ablation, or their synergistic combination. Therefore, the results of this study need to be further verified through prospective studies. Thirdly, this study included some non-treatment-naïve patients who had received other treatments before the start of the study, which made the interpretation of the research results more complex. Furthermore, univariate and multivariate analyses did not identify variables with a significant impact on OS, possibly due to the small sample size. Based on this, we are conducting a prospective randomized controlled trial (ChiCTR2300067319), using traditional TACE combined with ablation therapy as the control group, to investigate whether the combination of H101 and TAE sequential ablation therapy can reduce the recurrence of HCC and provide stronger treatment outcomes, thereby identifying the population that may respond effectively to H101 immunotherapy.

## Conclusion

HI01 combined with TAE followed by sequential thermal ablation is safe and effective in the treatment of HCC. In observational analyses, this therapeutic approach is associated with more favorable outcomes in patients with a maximum tumor diameter of ≤ 3 cm, AFP, or in BCLC stage B. This approach provides a new local treatment option for HCC. However, due to the limited number of current studies, future research with larger sample sizes and higher levels of evidence is needed to confirm these findings.

## Data Availability

The raw data supporting the conclusions of this article will be made available by the authors, without undue reservation.
